# ‘HIV ended up in second place’ − prioritizing social integration in the shadow of social exclusion: an interview study with migrants living with HIV in Sweden

**DOI:** 10.1186/s12939-022-01783-5

**Published:** 2022-12-06

**Authors:** Faustine Kyungu Nkulu-Kalengayi, Anne Adhiambo Ouma, Anna-Karin Hurtig

**Affiliations:** Department of Epidemiology and Global Health, Umeå University, SE-901 87 Umeå, Sweden

**Keywords:** Migrant/immigrant, Ethnicity, Intersectionality, HIV/AIDS, Language barrier, Multiple discrimination, LGBTQI-person, Racism, Xenophobia, Intersectoral collaboration, Sweden

## Abstract

**Background:**

Migrants are overrepresented among people living with HIV in Sweden as they often face conditions that increased their risk and vulnerability for HIV/STI infections prior, during or after migration. Yet, there is limited research on their experiences and perceptions of living with HIV in the Swedish context. This study aims to explore migrants’ experiences of living with HIV in Sweden.

**Methods:**

This is a qualitative study based on in-depth interviews with 13 migrants from 11 countries living with HIV in Sweden. Interviews were analysed with thematic analysis using an intersectional perspective to explore the interactions of multiple social identities such as ethnicity, socio-economic status, gender, age, and sexual orientation that shape an individual’s or group’s experiences.

**Results:**

The analysis resulted in a main theme: ‘Prioritizing social integration-HIV ends up in second place’, which is based on four subthemes: ‘Better opportunities in the new country than what the home country could offer’, ‘Better conditions for LGBTQI people than in the home country’, ‘Navigating a new system: linguistic and bureaucratic challenges’ and ‘Feeling like a second-class resident: racism, xenophobia and multiple discrimination’. The results suggest that migrants living with HIV in Sweden experience social integration as a greater challenge than HIV infection. Although the new country offers opportunities for better living conditions, many participants described being challenged in their daily life by linguistic and structural barriers in their encounters with public services. They are facing multiple discrimination simultaneously as migrants due to their multiple and intersecting identities (e.g. being non-white, foreigners/foreign-born and non-Swedish speakers), which is compounded by HIV status and thus limit their opportunities in the new country and too often result in an existence of exclusion.

**Conclusion:**

The study shows that most of the challenges that migrants living with HIV face are related to their status as migrants rather than HIV status, which is often not known by the public or authorities. These challenges are similar, but still differ depending on social position, previous experiences, time since arrival and since diagnosis. This emphasizes the importance of both intersectional, intersectoral and multisectoral approaches to address reported issues.

## Introduction

In recent decades, the morbidity and mortality related to human immunodeficiency virus (HIV) infection and/or acquired immunodeficiency syndrome (AIDS) have drastically declined globally due to improved and increased access to effective antiretroviral (ARV) therapy, which has resulted in an increase in the number of people living with HIV and AIDS [1]. Recent statistics in Sweden have also shown a decreasing trend in the number of newly diagnosed cases. Nowadays, more than 8,100 people are living with HIV (PLWH) diagnosis in Sweden, of whom just over 95% are under ARV treatment and over 95% of these have well-managed treatment regimes [2]. However, available statistics show disparities in reported HIV infection cases, access to HIV care, treatment and support, AIDS-related deaths between and within countries and continents. Some groups of the population are more vulnerable than others. For instance, migrants have been identified as a particularly vulnerable group in both the United Nation’s Declaration on AIDS 2021 [3] and the Swedish National Strategy against HIV and AIDS and certain other communicable diseases [4].

Migration is considered as a social determinant of migrant health as it cuts across existing social determinants, adding a particular social dimension and exacerbating other determinants. The conditions surrounding the migration process have a negative impact on the health of migrants in general [5] and play an important role in exposure to sexual risk and sexual risk-taking behaviours [6]. According to the European Centre for Disease Prevention and Control (ECDC) report, migrants made up approximately 12% of the population of the European Union (EU) countries but accounted for 44% of newly reported HIV cases in 2019 [7]. This is considered to be the result of the higher prevalence of HIV in their countries and an increased vulnerability of migrants due to the conditions surrounding the migration process [8]. Similar patterns have been seen in Sweden where migrants make up about 20% of the Swedish population but approximately 70% of new HIV infection cases being reported each year [2] and about 65% of PLWH in Sweden are foreign-born [9]. By the end of 2021, around 8,289 people living with HIV were being treated in Sweden, of which 61% were men and 39% were women. The majority were born abroad (64%), one third of them were born in sub-Saharan Africa (36%), 11 percent were born in Southeast Asia, mainly in Thailand. An equal proportion of five percent were born in Western Europe (5%) and Latina America and Caribbean (5%) followed by North America (4%). The remaining six percent were born in Eastern Europe and Central Asia (3%) and Southeast Asia (3%) [10]. Migrants are thus overrepresented among PLWH in Sweden and are one of the groups identified as vulnerable to HIV and other sexually transmitted infections (STIs) according to the Swedish National strategy against HIV and certain other communicable diseases [4].

### The legal context of HIV and migrants’ entitlements and access to public services in Sweden

HIV is classified as a public health threat in Sweden according to the Communicable Diseases Act (2004: 168). Consequently, coercive provisions of this Act can be applied to people who are living or are suspected to be infected with HIV. These include, among others, the obligation for PLWH to seek care, participate in contact tracing and follow the rules of conduct provided by attending doctors in order to prevent further transmission of the disease. These also include the obligation to inform and protect all persons who are at risk of being exposed to the disease, such as sexual partners and health-care professionals. However, amendments have been made to this act in 2013, allowing the attending doctor to exempt PLWH on ARV therapy who achieved and maintained an undetectable level of HIV in their blood also known as U = U from the legal obligation to disclose their HIV status [11]. This act also guarantees the right to access HIV testing, care, support and treatment free of charge for all [12]. On the other hand, the Swedish Discrimination Act (Law: 2008: 567) protects individuals against discrimination and promotes equal rights and opportunities in the society regardless of age, disability, ethnicity, gender, sexual identity or expression, sexual orientation, religion or other belief. The law prohibits discrimination in the public services, working life and schools [13].

There is no universally accepted definition of the term migrant. The United Nations Department of Economic and Social Affairs (UN DESA) defines an international migrant as any person who changes his or her place of usual residence [14]. In Sweden, except for Nordic citizens to be registered as a migrant, a foreigner should be in possession of a residence permit for at least 12 months. Citizens from the European Union/European Economic Area should meet requirements for right of residence through work, studies or with sufficient means.  After registration, each individual is assigned a unique personal identity number (*Personnummer*) by the Swedish Tax Agency according to § 4 (2021: 375) for their identification with authorities and access to health care and other public services [15]. The processing time varies and can be up to 18 weeks or longer. If you need care while waiting for the Swedish Tax Agency’s decision on your registration, you must pay the full cost of your care even if you have a residence permit.

Foreigners who have sought protection in Sweden (according to the 1951 Geneva convention on refugee status or for humanitarian reasons) and are awaiting a decision on their asylum claim (i.e. asylum seekers and other migrants staying in Sweden without the required permit (undocumented/irregular migrants) are entitled to care that cannot be postponed in accordance with the Acts (2008: 344) and (2013: 407) respectively. When asylum seekers seek care, they must present a special card called the LMA (*Lag* *om mottagande av asylsökande*: the law on the reception of asylum seekers) card or a receipt that the Swedish Migration Board has received their asylum application.

Furthermore, according to the Swedish Language Act (*Språklag* 2009: 600), Swedish is the official language (§ 4), in other words the common language of the Swedish society (§ 5) [16]. However, migrants and other persons who do not speak Swedish or have difficulty understanding information in Swedish are guaranteed the right to an interpreter while in contact with public services, including health and medical care and translation of documents in accordance with the Public Administration Act § 13 (*Förvaltningslagen* 2017: 900) [17]. Both interpreters and health providers are legally and professionally bound by secrecy [18].

### The lived experiences of people living with HIV in Sweden

Research on the experiences of migrants living with HIV in Sweden is very limited. The first Swedish comprehensive study on the experiences of PLWH in Sweden, conducted in 2013 [19], indicated that participants generally rated their quality of life as high. They were also satisfied with their physical and mental health as well as with relationships with friends, potential partners and family life. However, it was not possible to get a clear picture of how migrants living with HIV in Sweden assessed their quality of life. A subsequent qualitative study in Sweden, however, suggested that they experienced loneliness, fear of disclosing HIV status, stigma and lack of a social network [20]. Another interview study that aimed to describing the experiences of migrants living with HIV and their encounters with the Swedish health-care system, showed that they appreciated free access to ARV, but required more time with their doctors. They also described experiences of discrimination within the health-care settings outside the infection clinics [21]. On the other hand, an interview study on the sexual and reproductive health and rights of women living with HIV in Sweden revealed wide variations in experiences in relation to sexual and reproductive rights. It was, however, difficult to ascertain whether the stigma and discrimination experienced by migrant participants in this study were related to their migrant or HIV status or both [22].

Most migrants living with HIV in Sweden have different backgrounds and come from different countries in sub-Saharan Africa and South-East Asia. Few studies have simultaneously included migrants from different parts of the world, with different backgrounds, while exploring how these multiple identities, migrant and HIV statuses, interact and affect their lived experiences. Understanding the life experiences of migrants living with HIV thus requires an intersectional perspective that draws attention to the simultaneous and mutual effects of different social positions/markers of ‘identity and vulnerability’ that shapes these experiences. These include among others, ethnicity/race, gender, sexuality, class, disability, religion, or nationality [23]. In addition, migrant status has been identified as one of these axes of differences, which together with other categories, shapes disadvantages, privileges, health outcomes and access to available services in receiving countries [24, 25]. The intersectionality theory acknowledges and integrates the existence and fluidity of these multiple and combined identities that influence and are influenced by social structures of power and the policies that produce them. It does not consider these social identities as separate categories, but rather as mutually established systems of oppression and marginalization that work simultaneously to produce inequalities between groups and individuals [26]. Thus, we adopted an intersectional perspective to analyse how migrants’ multiple social identities (based race/ethnicity, nationality, class, gender, age, sexual orientation as well as migrant and HIV status) interact to shape their individual’s or group’s experiences within the Swedish context.

## Methods

### Aim and study design

This study aims to explore migrants’ perceptions and experiences of living with HIV in Sweden with a focus on their life situation and living conditions. The following research questions guided data collection and analysis:

1. What are migrants’ experiences of living with HIV in Sweden?
2. How do migrants living with HIV experience their life situations and living conditions in Sweden?
3. What are the challenges faced by migrants living with HIV and how do they deal/cope with these challenges?
4. How do these experiences of living with HIV intersect with other categories such as gender, ethnicity/race, sexual orientation, and immigration status?

A phenomenological design was used as this approach is appropriate for studying what an experience means to a particular group of people and how they experience it [27]. This is a qualitative interview study, which is well adapted to understand a phenomenon or situation from the participants’ perspectives by giving them an opportunity to express in their own words, different life experiences and lived situations [28].

### Recruitment and selection

Recruitment took place between June and November in 2021. We strived for a diversity in terms of age, gender, sexual orientation, education, civil/marital status, socio-economic status, place of residence and country/region of birth**.** Inclusion criteria were: being a migrant, i.e. having moved from another country to Sweden as a teenager or adult; aged 18 years or older; and diagnosed with HIV.

Participants were recruited via the Swedish Public Health Agency’s contact persons and partners in various regions, non-governmental organizations supporting PLWH, migrants and lesbian, gay, bisexual, trans, queer and intersex (LGBTQI)-persons, infectious disease clinics and through advertisements on social media (e.g. Facebook). A flyer in Swedish and an information letter about the study, including the research group’s contact information, were sent to the above-mentioned organizations/institutions directly or with the help of the Swedish Public Health Agency. Thereafter, those who agreed to disseminate information about the study received the flyer in nine different languages (Arabic, Dari, English, French, Tigrinya, Spanish, Swedish, Swahili and Somali) for dissemination via social media, mailing lists, newsletters, or noticeboards and distribution to migrants in the target group (i.e. PLWH). The poster/flyer also contained contact information for the research group to allow potential participants to get in touch by phone or email. Upon contact with the researcher, they received additional information about the study and those who were willing to participate agreed on the date, time and place for the interview with the researcher.

### Positionality statement

The first and second authors are migrant scholars with expertise in research on issues related to HIV/STI, migration, health and cultural diversity. They are multilingual/polyglot and have been residing in Sweden as migrants for decades, which allowed them to relate to participants, build trust and make study participants feel comfortable to share their lived experiences in a way they felt could best express their thoughts and feelings. However, they do not represent all migrants living in Sweden even though they share some common characteristics. The third author is a native Swede with expertise in health systems research including HIV prevention in Sweden and low- and middle-income countries. The three authors worked as a team with regular meetings throughout the study to discuss and ensure the study was guided by their expertise as well as their insider and outsider perspectives.

### Participants

The 13 participants originated from 11 countries. All participants had non-European ethnic origins, except for one who came from south-eastern Europe. One of the 13 participants abstained from answering the question of sexual orientation. Table 1 below shows the sociodemographic characteristics of participants.

**Table 1:** The sociodemographic characteristics of participants

Characteristic	Categories	Number
Region of birth	Sub-Saharan Africa	6
	Central and southwest Asia	2
	Latin America	3
	Southeastern and Western^a^ Europe	2
Gender	Woman	4
	Man	7
	Queer (Genderfluid)	1
	Trans (Man to woman)	1
Sexual orientation	Heterosexual	6
	Homosexual including lesbian	6
	Do not want to answer	1
Age	20-29 years	4
	30-39 years	4
	40-59 years	5
Level of education	Lower secondary school or less	2
	Upper secondary school	3
	Post-secondary school	8
Civil or marital status	Single	9
	Partner	3
	Multiple partner	1
Religion	Christian	7
	Muslim	3
	Atheist	3
Children	Yes	4
	No	9
Live with	Alone	6
	Partner	2
	Friends	2
	Children	1
	Group or employer’s accommodation	2
Residence permit	Yes	10
	No	1
	Awaiting decision/interview	2
Time since arrival	Less than one year	1
	1-5 years	6
	6-10 years	3
	11-20 years	1
	21-25 years	2
Occupation	Work	7
	Study	2
	Jobless	2
	On disability pension	2
Time since diagnosis	0-5 years	2
	6-10 years	5
	11-20 years	3
	21-30 years	2
	Do not remember	1
Region of residence in Sweden	Central	5
	Northern	5
	Southern	3

^a^Overseas ethnic origin

### Data collection

The interviews were individual and thematic. Each interview began with a structured interview section with background questions about, for example, gender, sexual orientation, age, education, country of origin and time since diagnosis. Thereafter a semi-structured thematic interview guide was used. Topics covered by the interview guide included: life situations, challenges and needs, coping strategies and perceptions and experiences of accessing social and other public services**.** The interview questions were adapted based on the participants’ life situation and on what they narrated.

The interviews were conducted by the first author (FKNK). A total of 13 interviews were conducted between June and November 2021. Three interviews were conducted via Skype (telephone) and nine via Zoom, of which seven were video interviews. Only one interview was conducted face-to-face at the research group institution. Each interview lasted between 46 and 96 min. The interviews were conducted in Swedish (*n* = 4), English (*n* = 6) or Arabic (*n* = 1), Tigrinya (*n* = 1) and Amharic (*n* = 1) with the help of interpreters. The thematic part of each interview was recorded after approval from participants who provided their consent before the interview started. After each interview, anonymous audio recordings/audio files were sent to an external professional party for transcription. After transcription, all interview material was listened to and checked to ensure that transcription and interpretation were as verbatim as possible. The so-called ‘analytical saturation principle’ has been applied, which means that data collection was ended when themes in relation to purpose were repeated in the interviews and new interviews would not add new content [29].

### Data analysis

The interview transcripts were analysed with thematic analysis using an inductive approach with the aim of identifying recurring themes/patterns in the informants’ stories. Braun and Clarke argued that even though one approach tend to dominate, coding and analysis actually often uses a combination of both inductive and deductive approaches. According to them, it is not possible to be purely inductive or deductive, as scholars always bring something to the data when analysing it, and they hardly completely ignore the data themselves when coding for a particular theoretical construct. They further described an inductive thematic analysis as “often experiential in its orientation and essentialist in its theoretical framework, assuming a knowable world and ‘giving voice’ to experiences and meanings of that world, as reported in the data”. Thus, an inductive approach prioritises participant or data based meaning [30]. The analysis process was based on the following six steps described by Braun and Clarke [30]:

1) All researchers read the interviews to become familiar with the data. Thereafter, a discussion was held within the research group on preliminary thoughts and interpretations.

2) Two researchers read and coded each of the 13 interviews.

3) The coded interview material was sorted under broader emerging themes, which were then discussed within the research group to identify potential themes and subthemes.

4) The themes and subthemes developed in step three were reviewed and a thematic map was created based on a compilation of a final list of codes, subthemes and themes.

5) Final themes were defined and named.

6) Compilation of findings including description of themes 

## Results

After the analysis, a main theme: ‘Prioritizing integration − HIV ends up in second place’ and four subthemes: 1) Better opportunities in the new country than those that the home country could offer; 2) Better conditions for LGBTQI people than in their home country; 3) Navigating a new system: linguistic and structural barriers; and 4) Feeling like a second-class resident: racism, xenophobia and multiple discrimination were generated. The main theme, ‘Prioritizing integration − HIV ends up in second place’, described the participants’ experiences of life with HIV, where the biggest challenge is social integration in a new country, which took more time and effort than the HIV infection itself. In this regard, the challenges posed by HIV seemed to come in second place, after those related to settling in a new country.

Participants were grateful and described how fundamental rights and the welfare system in Sweden provided opportunities for better living conditions and care for migrants living with HIV than those that their home countries could offer. At the same time, they faced complex challenges in the form of linguistic and structural barriers, and multiple forms of discrimination simultaneously, mainly due to their race/skin color (racism), ethnicity (ethnocentrism), nationality (xenophobia), which limited their opportunities in the new country. These challenges were considered to be more problematic than the disease itself, although it was still present in their minds. Migrants thus prioritized, over other matters, learning the language and how to navigate the various public services involved in the integration processes in the new country. One participant expressed it this way:

But then when I came here, it was not HIV that was the most important/urgent thing, rather it was learning the language, understanding how the society works; “what is it like to live here?”, and so on. HIV ended up in second place, but it was there anyway, along with these thoughts [P1].

These experiences were also influenced by participants’ past experiences, social identities based on age, gender, sexual orientation, education, ethnicity and economic status, religion as well as the migration and health policies, legislation and practices within the Swedish context, resulting in varied experiences.

### Better opportunities in Sweden than those that the home country could offer

Participants described better living conditions for people living with HIV as well as access to better HIV care and treatment in Sweden than in their home countries. Several participants had experienced strict rules, degrading treatment, prejudice and social stigma in their home countries, which often resulted in feelings of shame and discomfort. They felt that compared to experiences in their country of origin, Sweden offered opportunities for a more normal life, with entitlements to fundamental rights, without HIV-related discrimination. Participants were grateful for the existing social safety net in Sweden. This included housing, various types of social benefits and health care. One participant expressed gratitude for a good and safe welfare system in Sweden that did not exist in his home country and declared**:** ‘In Sweden, all people are guaranteed their own accommodation/housing, Sweden guarantees everyone the right to food, heat, water. Yes, the basic stuff you need to live. In my home country, one has to fend for oneself’ [P4].

Some participants highlighted how legislation in Sweden was protecting PLWH and guaranteing their human rights such as the right to work and education, as well as the right not to be subjected to inhumane and degrading treatment. Such legislation did not often exist in their home countries, where they witnessed that those infected with HIV were arrested, detained, dismissed or suspended from universities and working within the healthcare and public sectors:

I think the laws in Europe are such that your HIV diagnosis is not going to affect your access to work or university in the country where you live, or like wherever you work.... But in my country, you cannot even go to university with HIV. [P8]

Participants were also grateful for access to effective and comprehensive HIV care and support in Sweden, regardless of their legal status. Some reported that the medicines they received in their home countries were old, of poor quality, ineffective and had many side effects. Some were able to switch medicines after arriving in Sweden:

I took it [ARV] every day according to the prescription, but after I moved to Sweden, I had 3000 viruses [HIV copies per milliliter of blood] in my blood, and then when I received another treatment and medicines in Sweden, it dropped to 150 after a few months, you see the difference …big difference. [P12]

Participants also appreciated the availability of care and the opportunity to be able to communicate with treating physicians regularly as well as having access to HIV care regardless of whether they had a residence permit or not:

The first thing is, I’m not even legal in Sweden. I have no insurance. And I’m not even a student. Not working. And like, I have no rights, basically. But still, I get the same level of [HIV] treatment as everyone gets. That's the best thing that happened to me. [P8]

In contrast, they considered that HIV care was difficult to access, of poor quality and discriminatory in their home countries, where staff seemed to lack knowledge and had negative attitudes and prejudices against PLWH. Some participants described how they felt exposed in their home country because HIV services were not integrated or easily accessible. One participant portrayed the situation in his home country and said: ‘If you lived outside the capital, then you had to travel there every month to get medication. It was not even certain that you would receive medication every month either’ [P11].

### Better conditions for LGBTQI people than in the home country

Participants belonging to the LGBTQI group who came from countries where homosexuality was illegal and where homophobic attitudes were prevalent experienced better conditions for living with HIV in Sweden than in their home countries. They described how they were forced to hide their sexual orientation and gender identity as this exposed them to dual stigma and discrimination as PLWH too. As a result, they were subjected to persecution, imprisonment, degrading treatment, hatred and death threats from relatives and society. They pointed out that current legislation in Sweden made it a safe and secure country where LGBTQI people could live a normal life and access HIV treatment without discrimination, as highlighted by one participant: ‘Coming to Sweden as a gay person, I feel safer. Nobody attacks me. You feel safe on the streets’ [P7].

Another participant recounted how she was subjected to sexual violence as a punishment for LGBTQI activism in her home country:

So, we were demonstrating when they were going to pass the law of killing all LGBTQ persons in my country. So, they got us and they arrested us and every day they would...they used to send in men in the night to rape us in these cells. So, it was a very horrible experience. [P2]

The link between HIV, sexuality and religion resulted in stigmatization and marginalization of LGBTQI-persons, both in society and within families in home countries. Getting an HIV diagnosis was considered shameful since it was associated with prostitution and homosexuality, as voiced by one participant: ‘When you belong to the LGBT community in my country. “The result is that you always get HIV at the end”, that’s what they say’ [P9]. Therefore, some preferred to hide their sexual orientation. Another participant described his initial reaction to his HIV diagnosis:

My mind went blank [when I was diagnosed], I was very scared, especially ashamed, not to die, but to show that I am gay, because in the beginning it was [HIV] a disease for prostitutes and homosexuals. [P4]

Their experience of HIV care in their home countries as LGBTQI persons was that they did not get the care they required because health-care professionals focused on their sexual orientation instead of their care needs. Some completely avoided seeking HIV care in their home country due to fear of discrimination and the perceived risk of being reported to the police. They were not convinced that medical staff were bound by professional secrecy obligations in relation to police authorities and worried that their sexual orientation would be revealed as illustrated in the quote below:

And I was being honest, I told them [health-care staff] that I had sex with men. And they gave all the reports to police…So, people don’t go to get treatment for their HIV because of that. Because they’re afraid that they’re gonna be jailed for being gay. [P8]

### Navigating a new system: linguistic and bureaucratic challenges

Participants described not only the opportunities that opened up for them in Sweden but also the challenges they experienced. They talked about linguistic and structural barriers, lack of information about their rights, and a cumbersome bureaucracy that was difficult to navigate. Participants described various challenges that they faced in their daily life with regard to access to employment or available services including health-care services. They argued that their HIV status was not often an obstacle, rather the actual challenge lies in settling into a new society, as stressed by one participant: ‘But if you have your little job and can take care of yourself, will HIV be a problem?**…** No, you will be taking your drugs and HIV will not be something that will bother your life anymore’ [P9].

Language was considered as one of the biggest barriers to social integration as it restricted access to information about vital public services, individual rights as well as communication and social interaction in the new country. Participants pointed out that all available information was in Swedish, while sometimes in English, which made it difficult to understand. One participant explained: ‘Well, yeah. That everything is just in Swedish. It is really hard to know how to use it. They should also provide information in English or in other languages’ [P3]. Another participant recalled his experience as a newcomer: ‘Yes, then language barriers and lack of knowledge about rights, I think so, absolutely. I had almost no control at all over what one was allowed to do and which opportunities one had’ [P1].

Some participants reported difficulty communicating in different contexts due to language barriers. Participants appreciated access to interpreters, but felt uncomfortable about expressing themselves through interpreters, and not quite safe to have one in the room who could sometimes be an acquaintance or even behave unprofessionally, as stressed by one participant: ‘Yes, there was no interpreter on site but an interpreter by phone. I did not want to meet the interpreter in person’ [P10]. Those who had learnt the language, gained knowledge about vital public services and their rights, felt equipped to express their thoughts, that the challenges they previously encountered had disappeared and that they had better prerequisites for socialization and integration in the new country.

I think I have the tools that I need, and that’s because I understand how the society works, I know my rights and I can speak Swedish ... it’s like, yes, some of the challenges simply disappeared. [P1]

Besides language barriers, participants described structural barriers that made it difficult for them to navigate the Swedish system and utilize available services. Structural barriers were considered a major problem for accessing available services. It was difficult to know where to turn and what you were entitled to. A personal identity number and residence permit were considered as imperative, as they determined to a great extent what one was entitled to or not in the Swedish system. One participant said:

Arbetsförmedlingen (*Swedish Public Employment service*) said: ‘you are registered but to work with you, you must have a personal number’. Försäkringskassan (*The Swedish Social Insurance Agency*) were also saying the same. Yet the number had not come. You can’t go to SFI (*Swedish for Immigrant school*), get a job, if you don’t have a personal number. You can’t really access many things here. [P2]

Accessing available services was considered to be complicated and time-consuming and this was compounded by bureaucracy, paperwork and digitization. Most contacts were made through phones or emails with different types of booking systems, all of which could be difficult to navigate when one was in urgent need of help:

What I don’t like here [in Sweden] is most people are on the telephone, media, you see. It is not that easy to meet someone to talk face to face. Yeah, that is what I don’t really like here because eh, yeah: ‘contact this number, contact this mail, contact this, contact this, contact this’, [P9]

### Feeling like a second-class resident/person: concurrent discrimination on multiple grounds

Participants described various forms of discrimination, based on their race or skin color (racism), ethnicity (ethnocentrism), language (language oppression) and nationality/nativity (xenophobia), that they experienced in their encounters with public services, as well as in daily life at the workplace and in the society. This was said to lead to minority stress, a feeling of inferiority, exclusion and loneliness. Several said that they were unfairly and ill-treated in various social contexts. Such discrimination was perceived as frustrating and made them feel like a second-class resident. One participant said: ‘Yeah, you are a second-hand person, second-hand citizen. They treat you like a piece of shit [laughs], but when it comes to Swedish people, they are extremely polite and understanding. It is annoying and frustrating’ [P7].

Several participants stated that the Swedish government was maintaining a facade of a welcoming country, but behind that facade hid a racist society that was not ready to accept ethnic/racial diversity. One participant reflected: ‘But sometimes it feels a bit upside down… Sweden receives a lot of people here and says: “everyone is welcome”, but the public is a bit racist. Eh, so sometimes you do not know what to think’ [P5].

Racism was also experienced in the public space. One non-white participant described such an event during a trip:

One time I was in a train and a guy said: ‘monkeys, monkeys’ in front of me and my other black friend. ‘Monkeys’ and he was even doing actions. Then I said: ‘Let’s not give him attention, let’s stand up, leave and go to another seat. And then he came and followed us and said: ‘monkeys’ [P2].

It was, however, common for participants to state that the discrimination and distinctive treatment that they were subjected to often took a more subtle form. The participants believed that society harbors feelings of, or there was a certain irritation towards, migrants in the society that made them even more vulnerable in everyday life. This was often expressed not through words but in the form of micro-aggressions, demeaning behavior and a cold shoulder. One participant said: ‘Maybe someone cannot tell you straightforward, but they may ignore you and you see that there is something happening because of how you look or… It happened to me’ [P6]. This contrasted with the cultures of their home countries, where people often expressed themselves bluntly when they disapproved of someone or something. They considered Swedes to be politically correct.

According to participants, the grounds for discrimination varied but it was mainly due to their ethnic/racial origin, and not specifically to their HIV status. They argued that few people knew about their HIV status in different social contexts. They, therefore, gave an account of discrimination that in many ways strongly affected their life situation. Many suggested that migrants constituted a vulnerable group that was discriminated against consciously or unconsciously, not only by individuals but also by societal institutions, regardless of where they came from, their education level, or whether they could speak the language or not. One participant argued that as long as you have another skin color (non-white) and a foreign name, you will always be considered as a migrant regardless of how long you had been living here or whether you were born in Sweden or not:

So, I do not think I will stop being a migrant here, as long as my name is not ‘Johan Andersson’ or as long as I do not look like what a normative Swedish person could look like. Also, the language .... It would be better [if I change my name] but I still do not think ... No, I do not think it will work out... [P11]

Some participants felt that their migrant status in combination with their HIV status resulted in or led to double or indeed triple discrimination, as stated by this participant:

Sometimes it’s like being an immigrant and HIV-positive person puts me in a more vulnerable position because you get discriminated on being an immigrant level and then discrimination on being HIV-positive level. Then, you are discriminated in combination of those two factors. So, it is like, being discriminated three times. [P7]

However, the experiences of discriminatory treatment and how it changed over time varied among participants. They described different experiences based on their skin color, ethnic origin, gender, civil status and language skills. One participant, MSM, with a light skin color who had lived in Sweden for many years reasoned: ‘I am fluent in Swedish, which means that most of the times I am not even perceived as a migrant just because I am white and speak good Swedish’ [P1].

Participants also found different ways to cope with their experiences. Some mentioned that they had survived many traumatic and stressful events, which had made them stronger and that was why they could handle discriminatory treatment that they were subjected to in Sweden. Others said that they got used to it and no longer let it affect them. They described different coping strategies that enable them to move forward. Some participants shared their experiences:

I have been operated on a thousand times. In my life, I have been beaten by two men that I had a relationship with, I have been raped… but still had a great strength in me, that I can stand up… I stand up and become a human being again. [P5]

Yes, in the past I used to be a bit sad and angry [when I did not get help or was discriminated against]. But now, I just endure it. [P10]

## Discussion

This study provides knowledge about migrants’ own perspectives and experiences of living with HIV in Sweden, with a focus on living conditions. The results suggest that migrants living with HIV in Sweden prioritize social integration, which they experience as a greater challenge than HIV which often ends up in second place. Even though the new country offers new opportunities, many participants are challenged in their daily life by linguistic and structural barriers in their interactions with public services. They are facing multiple forms of discrimination simultaneously (intersectional discrimination) as migrants due to their multiple and intersecting identities (e.g. being non-white, foreigners/foreign-born and non-Swedish speakers), which is compounded by HIV status, and thus limit their opportunities in the new country and too often result in an existence of exclusion.

An important finding of this study is that the life situation, experiences and challenges revolve primarily around being a migrant. From an intersectional perspective, migrant status is considered as a social category or one of the axes of differences, but it is argued that ‘migration experience’ is not a universal and homogenous experience, but rather that there are diverse migration experiences that are shaped by the particular dynamics of ethnicity/race, gender, class/socioeconomic position, geography, and other categories of difference such as HIV status [25]. Migrant status has been identified as social identity category and one of the axes of differences, which together with other categories, shape disadvantages, privileges and health outcomes and the extent to which one has access to services [24, 25]. For instance, while a white gay male/MSM long term migrant who is fluent in Swedish described being privileged, a non-white lesbian recent migrant with limited proficiency in Swedish reported disadvantages including discrimination, difficulty in accessing support and services.

Previous international studies [31, 32] have also shown significant differences in experiences among migrants based on several factors, although they have common experiences related to being migrants [31–36]. Previous Swedish studies also show challenges faced by migrants in terms of health inequalities due to different living conditions and experiences, which place them in different social positions with different degrees of vulnerability. [20–22].

Nevertheless, the study shows that many participants feel that they have better opportunities in Sweden compared to their home countries, in the form of varied support, including housing and other social support. For instance, Sweden’s enforcement of LGBTQI people’s rights and free access to HIV treatment and care for all PLWH (to control the spread of HIV infection) have been highlighted as life changing and that have created safety for most/many participants. This provides opportunities, particularly to those who were LGBTQI-persons to live a full life and to be themselves. This is an important contribution to the literature as previous studies are limited in this area [37]. In general, the participants experience a ‘fairer society’, less stigma and taboo surrounding HIV, homosexuality and adapted support on arrival in comparison with the country of origin. Stigmatization in relation to sexual orientation varies depending on the context. A previous north American (USA) study has shown that sexual orientation is a stigmatizing identity that interacts with other stigmatizing identities (ethnic origin and legal status) that affect employment opportunities and retention in HIV care [38].

The study further reveals that the needs and challenges that migrants living with HIV meet mainly revolve around social integration and language is an important challenge for access to information, available services and communication. Swedish authorities/public services are obliged to communicate in Swedish, urged to use interpreters and translate information in appropriate languages for those who do not speak or understand Swedish [17]. However, there are challenges in the triad communication, while most available information is only (provided) in Swedish, which creates a language barrier for participants that leads to complex challenges. The study shows difficulties in gaining access to available information about Swedish society that is provided in a language that is not as well understood, lack of information about Swedish society in general and how it works. On the other hand, if one acquires language skills and receives support, it enables easier access to information, which makes it easier to manage the disease and settle/integrate oneself in the new society. Language is essential for navigating the new system, including finding work and creating social relationships in the new country, as well as claiming one’s rights. Previous international [39, 40] and Swedish [41, 42] studies have also identified language as an important barrier to access to available services, information and communication. However, the challenges faced are not limited only to language as it also includes structural barriers. Previous studies have also shown that complex structural barriers affect access to housing and employment, which are important determinants of health [38, 43–45]. The main obstacles are due to their multiple identities as migrants and people living with HIV. These include long waiting periods, lack of a health card [46], lack of a residence permit [36, 45] and work experience from the host country [45], unemployment [36] and work restrictions and bureaucracy [43] and fear of deportation [43, 47]. These challenges for integration in the new society affect their well-being and health.

Other challenges described by participants included simultaneous and multiple forms of stigma and discrimination at the interpersonal/individual, institutional and structural levels. This was not primarily on their HIV status but rather their multiple and intersecting identities as migrants [48]. They face discrimination on multiple grounds including their race/skin colour (racism due to being non-white), ethnicity (ethnocentrism due to non-Swedish ethnic origin), nationality or nativity (xenophobia due to being foreign-born or foreigners), language (language oppression due to limited proficiency in Swedish, e.g. being excluded from conversations). These distinct and specific forms of discrimination interact with one another and produce a compound dimension of disempowerment (intersectional discrimination). Although ethnicity is one of the seven grounds of discrimination covered by the Swedish Discrimination Act (2008:567) that prohibits discrimination, participants were mostly affected by discrimination related to their ethnic background, which leads to a sense of inferiority [13]. In addition, regardless of their HIV status, migrants are more likely to be socially marginalised because of multiple identities based on race, nativity, immigration status, ethnicity, and language through different processes including material deprivation, restrictive policies, cultural and linguistic barriers, discrimination and traumatic experiences. In addition, regardless of their HIV status, migrants are more likely to be socially marginalised because of multiple identities based on race, nativity, immigration status, ethnicity, and language through different processes including material deprivation, restrictive policies, cultural and linguistic barriers, discrimination and traumatic experiences. All of these factors operate simultaneously and make migrants one of the most vulnerable and marginalised groups of the population. Meanwhile the stigma related to their intersecting identities reinforce their vulnerability to discrimination based on their HIV status [5, 24, 48]. Several studies exist on how HIV related stigma and discrimination and racism in society create social and structural barriers and how these affect access to housing, employment, health and life in general among migrants living with HIV to produce inequalities [31, 36, 38, 44, 49].

Last, but not least, this study suggests that migrants living with HIV develop different coping strategies to deal with challenges surrounding the social integration processes. It reveals participants’ struggle against social exclusion and isolation over time and their experiences of resilience in order to live a normal life, which is also described in a previous study [50]. Some accounts indicate that they have had terrible experiences from their home countries, something that has *hardened* them and made it easier to cope when they meet with the social exclusion many migrants experience in Sweden.

### Strengths and limitations

There are both strengths and weaknesses with the study. One of the strengths of the study is that participants were recruited through different networks, civil society organizations and infection clinics working with PLWH resulting in a sample of people belonging to different age groups, having different marital status, gender identities, sexual orientation, level of education and legal status, being born in different continents, having lived with HIV and in Sweden for different lengths of time, which leads to a wide range of experiences and provides opportunities to adopt an intersectionality perspective in the analysis. Furthermore, the interviews were conducted by a researcher with previous experience of qualitative interviews as well as research on migration and HIV. Three experienced researchers were involved in the design of the interview guide and analysis. Two of the three researchers have a migrant background, with language skills that have been of great help in conducting and interpreting interviews with participants who had limited language skills in Swedish and English. The use of zoom/online and telephone interviews enabled the interviews to be conducted with participants from different geographical areas, despite COVID-19 restrictions in 2021, and to ensure anonymity by letting interviewees themselves choose which level of contact they wanted (video on or off / telephone).

However, we failed to recruit participants who were not in contact with HIV organizations through Facebook ads, despite several attempts, probably due to fear of social stigma. Another limitation may be that we have only managed to recruit one heterosexual man. The use of zoom/telephone interviews encouraged participation because participants could be anonymous and speak freely at the expense of interpreting body language. Finally, the use of interpreters helped address the language barrier, but it is not unproblematic, something that the researchers were aware of from previous experience.

### Conclusion

The study shows that most of the challenges that migrants living with HIV in Sweden face are related to their status as migrants rather than HIV status, which is often not known by the public or authorities. These challenges are similar, but still differ depending on social position, previous experiences, time since arrival and since diagnosis. This emphasizes the importance of both intersectional and multisectoral approaches, which consider the interplay between different social categories, such as gender, sexual orientation, ethnicity, socio-economic status, and migrant status through collaboration across various government agencies, non-governmental organizations and other relevant stakeholders to address the issues described in this study at different levels. These include, at the individual level: tailored information about available services, rights, existing legislation to facilitate social integration; at the institutional level: liability for the application of the Discrimination Act and improvement of interpreting and translation services; and at the structural level: intersectoral policy, collaboration and strategies.

## Data Availability

The datasets used and/or analysed during the current study are available from the corresponding author on reasonable request.

## Abbreviations

AIDS: Acquired immunodeficiency syndrome
ARV: Antiretroviral
COVID-19: Coronavirus disease 2019
Dnr: Registration number (Swedish: *Diarienummer*)
ECDC: European Centre for Disease Prevention and Control
HIV: Human immunodeficiency virus
LGBTQI: Lesbian, gay, bisexual, trans, queer and intersex -persons
LMA: The law on the reception of asylum seekers (Swedish: *Lag* *om mottagande av asylsökande*)
MSM: Men who have sex with men
PLWH: People living with HIV
SCB: Statistics Sweden (Swedish: *Statistik Centralbyrån*)
SFI: Swedish for Immigrants (Swedish: *Svenska för invandrare*)
STIs: Sexually transmitted infections
UNAIDS: The Joint United Nations Programme on HIV/AIDS
